# Deep Learning for Nasopharyngeal Carcinoma Segmentation in Magnetic Resonance Imaging: A Systematic Review and Meta-Analysis

**DOI:** 10.3390/bioengineering11050504

**Published:** 2024-05-17

**Authors:** Chih-Keng Wang, Ting-Wei Wang, Ya-Xuan Yang, Yu-Te Wu

**Affiliations:** 1 School of Medicine, College of Medicine, National Yang-Ming Chiao Tung University, Taipei 112304, Taiwan; jimmywang0504@gmail.com (C.-K.W.);; 2 Department of Otolaryngology-Head and Neck Surgery, Taichung Veterans General Hospital, Taichung 407219, Taiwan; 3 Institute of Biophotonics, National Yang-Ming Chiao Tung University, 155, Sec. 2, Li-Nong St. Beitou Dist., Taipei 112304, Taiwan

**Keywords:** nasopharyngeal carcinoma (NPC), deep learning (DL), magnetic resonance imaging (MRI), segmentation, convolutional neural networks (CNNs)

## Abstract

Nasopharyngeal carcinoma is a significant health challenge that is particularly prevalent in Southeast Asia and North Africa. MRI is the preferred diagnostic tool for NPC due to its superior soft tissue contrast. The accurate segmentation of NPC in MRI is crucial for effective treatment planning and prognosis. We conducted a search across PubMed, Embase, and Web of Science from inception up to 20 March 2024, adhering to the PRISMA 2020 guidelines. Eligibility criteria focused on studies utilizing DL for NPC segmentation in adults via MRI. Data extraction and meta-analysis were conducted to evaluate the performance of DL models, primarily measured by Dice scores. We assessed methodological quality using the CLAIM and QUADAS-2 tools, and statistical analysis was performed using random effects models. The analysis incorporated 17 studies, demonstrating a pooled Dice score of 78% for DL models (95% confidence interval: 74% to 83%), indicating a moderate to high segmentation accuracy by DL models. Significant heterogeneity and publication bias were observed among the included studies. Our findings reveal that DL models, particularly convolutional neural networks, offer moderately accurate NPC segmentation in MRI. This advancement holds the potential for enhancing NPC management, necessitating further research toward integration into clinical practice.

## 1. Introduction

Nasopharyngeal carcinoma (NPC) is a distinct head and neck cancer subtype originating in the nasopharynx, the upper region of the throat posterior to the nasal cavity [1]. Despite its rarity on a global scale, NPC exhibits a higher incidence in specific geographic regions, such as Southeast Asia and North Africa, likely attributable to a combination of genetic, environmental, and Epstein–Barr virus-related factors [2,3]. The early detection and accurate diagnosis of NPC are paramount for optimal treatment planning and improving patient prognosis [4]. However, the complex anatomy of the nasopharynx and the variability in clinical presentation make early detection and accurate diagnosis of NPC challenging.

In this context, magnetic resonance imaging (MRI) is the preferred imaging modality for the diagnosis, staging, and treatment planning of NPC due to its superior soft tissue contrast resolution compared to other imaging techniques, such as computed tomography (CT). MRI’s excellent contrast resolution allows for an accurate delineation of the primary tumor, assessment of local invasion, and detection of lymph node involvement. This detailed visualization of the tumor’s extent significantly enhances NPC’s staging and treatment planning by accurately outlining the tumor’s extent [5]. The capability of MRI to accurately delineate the extent of tumor invasion is crucial for precise staging, which is a critical determinant of therapeutic strategies [6]. Moreover, MRI offers the advantage of not exposing patients to ionizing radiation, making it a safer choice for repeated imaging during follow up and treatment response evaluation. The multiplanar imaging capabilities of MRI further enhance the assessment of tumor spread in various planes, providing a comprehensive understanding of the disease extent.

However, despite the advantages of MRI, the manual segmentation of NPC from MRI, a prerequisite for accurate tumor delineation, is a time-consuming and subjective process and is prone to inter- and intra-observer variability [7,8]. This variability can lead to inconsistencies in tumor volume estimation, staging, and treatment planning, potentially impacting patient outcomes. Therefore, there is a pressing need for automated, reliable, and efficient segmentation methods to overcome these limitations and improve the clinical management of NPC.

The advent of deep learning (DL) technologies has revolutionized the field of medical imaging, offering novel paradigms for automated image analysis. Deep learning models, particularly convolutional neural networks (CNNs), have demonstrated remarkable performance in image recognition, segmentation, and analysis tasks, surpassing traditional image processing methods in terms of accuracy and efficiency [9,10]. In the context of NPC segmentation, DL models have the potential to overcome the limitations of manual segmentation by providing rapid, accurate, and reproducible results [11,12].

Previous reviews have focused on either the segmentation of head and neck cancers in general [13] or NPC segmentation using both computed tomography (CT) and MRI [14]. This systematic review and meta-analysis aim to synthesize the current evidence on the application of deep learning for NPC segmentation in MRI. Our study offers several unique contributions to the existing literature on DL applications in NPC segmentation. First, we focus exclusively on MRI, providing a comprehensive analysis of DL performance for this specific modality. Second, our review includes the most recent studies from 2023 and 2024, ensuring up-to-date evidence. Third, we employ a novel three-level meta-analytic approach that accounts for all reported results across validation sets, revealing significant heterogeneity among independent datasets. This finding underscores the need for further research to explore the sources of variability and standardize DL model development and validation.

## 2. Materials and Methods

### 2.1. General Guidelines

This study maintained high methodological quality during its planning and dissemination phases in line with the Preferred Reporting Items for Systematic Reviews and Meta-Analyses (PRISMA) 2020 standards [15]. We followed the PRISMA guidelines closely, and the complete checklists are available in the Supplementary Materials (Tables S1 and S2). This research is registered with the International Platform of Registered Systematic Review and Meta-analysis Protocols (INPALSY), and the registration identifier is INPLASY202430120 [16]. As this systematic review and meta-analysis did not involve human participants, ethical approval and informed consent were not required, which was consistent with the guidelines for such studies.

### 2.2. Search of Databases and Selection of Eligible Studies

To select studies on DL applications for segmenting NPC in MRI scans, two reviewers (T-WW and C-KW) conducted a detailed literature review across PubMed, Embase, and Web of Science, covering all records up to 20 March 2024 (see Supplementary Table S2 for search details). The search strings were ((Nasopharyngeal Neoplasms OR Nasopharyngeal Cancer OR Nasopharyngeal Carcinoma OR Nasopharyngeal Tumors) AND (MRI OR magnetic resonance imaging OR MR) AND (segmentation OR contouring OR delineation) AND (deep learning OR convolutional neural networks OR CNN)) and are further detailed in Table S3. The process included title and abstract screening supplemented by manual searches to capture pertinent studies comprehensively. Any disagreements in study selection were resolved by consulting a third expert. We only included studies that applied DL for NPC segmentation in adult patients using MRI scans. Exclusions were made for non-MRI studies, retracted conference papers, Supplementary Materials, studies not addressing the research question directly, or those lacking necessary data for meta-analysis (e.g., missing standard deviation of Dice scores).

### 2.3. Data Extraction and Management

T-WW and C-KW collected key data from the chosen studies, including the study design, patient counts, and the number of series in training and testing sets. They also reviewed the sources of data, the validation techniques used for the models, and the standards for establishing reference values and indicators for ground truths. The documentation included MRI image specifics like magnetic field strength, sequences, and the manufacturer and model of the MRI equipment. The evaluation of the algorithms focused on their dimensions and types. This was accompanied by a detailed review of preprocessing methods, covering normalization, resolution resampling, data augmentation, and image cropping techniques. An extensive evaluation of the Sørensen–Dice coefficient was performed, highlighting its crucial role in assessing segmentation accuracy in these studies.

### 2.4. Methodological Quality Appraisal

Two established tools were used to evaluate the methodological quality of the studies: the Checklist for Artificial Intelligence in Medical Imaging (CLAIM) and the Quality Assessment of Diagnostic Accuracy Studies-2 (QUADAS-2) [17,18]. T-WW and C-KW conducted these assessments independently to minimize bias. Disagreements were resolved by consulting senior researchers and ensuring a consensus-based, rigorous quality assessment. This approach underscores the commitment to methodological precision and consensus in evaluating study quality.

### 2.5. Statistical Analysis

Two meta-analyses assessed the Dice scores reported by the studies. The first analysis selected the highest-performing algorithm when multiple outcomes were reported per study or when different studies used the same validation dataset. Median and interquartile ranges were converted to mean and standard deviation using established formulas [19,20]. A random effects model with restricted maximum likelihood was applied to accommodate study population heterogeneity [21], visualized through forest plots and assessed via sensitivity analysis (leave-one-out method) and subgroup analyses on variables like publication status [22]. The Q test quantified heterogeneity across studies, setting statistical significance at a *p*-value of <0.05. Heterogeneity levels were categorized by I^2^ values as trivial (0–25%), minimal (26–50%), moderate (51–75%), and pronounced (76–100%) [23]. To assess publication bias, Egger’s method for funnel plot asymmetry was employed, utilizing Stata/SE 18.0 for Mac [24].

The second meta-analysis explored DL algorithm performance variability across validation sets, addressing dataset reuse by comparing bi-level and tri-level random effects models, the latter clustering by dataset to mitigate mixed effects from validation reuse. The variance was assessed across three levels—datasets, repeated analyses, and study samples—using analysis of variance and Cheung’s formula [25]. Meta-regression [26] incorporated moderators like dataset splitting (train/test vs. cross-validation), the validation method (internal validation vs. external validation), MRI sequence (single vs. multiple), algorithm type (U-net, U-net variants vs. CNN), training size, and preprocessing techniques (intensity normalization, resolution adjustment, image augmentation, and image cropping). Statistical analysis was conducted with the metafor package in R, considering *p* < 0.05 as significant.

## 3. Results

### 3.1. Study Identification and Selection

**Figure 1:**
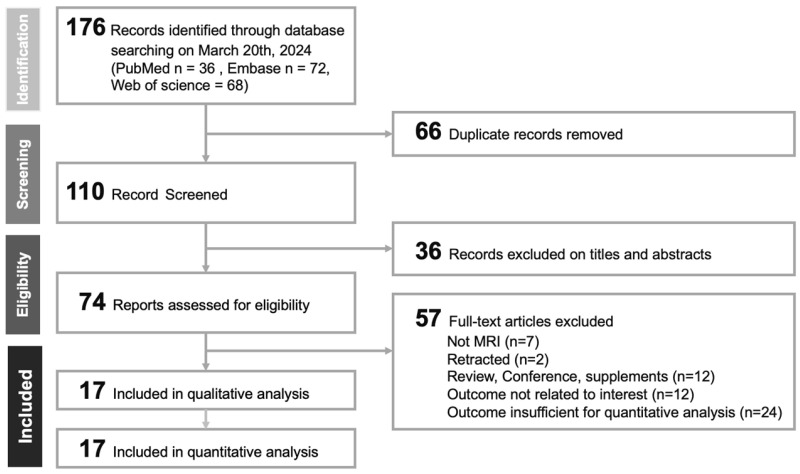
PRISMA flowchart for study selection.

The PRISMA diagram (Figure 1) illustrates the exhaustive search and selection methodology adopted in the present investigation. Initially, a comprehensive search was conducted across various databases from inception to 20 March 2024, yielding 176 studies, comprising 36 from PubMed, 72 from EMBASE, and 68 from Web of Science. After 66 duplicates were removed, 110 articles were further assessed using EndNote software. An initial review of titles and abstracts led to the exclusion of 36 articles, attributed to their irrelevance or lack of comprehensive detail. Further evaluation of the 74 full-text articles resulted in the exclusion of 57 articles [8,27,28,29,30,31,32,33,34,35,36,37,38,39,40,41,42,43,44,45,46,47,48,49,50,51,52,53,54,55,56,57,58,59,60,61,62,63,64,65,66,67,68,69,70,71,72,73,74,75,76,77,78,79,80,81,82] for various reasons, including the nature of the content being reviews, supplements, or conference abstracts; the absence of MRI application; retraction status; irrelevance to the scope of the current meta-analysis; or the inadequacy of reported outcomes for quantitative synthesis (refer to Table S4). This selection process culminated in the selection of 17 studies [11,12,83,84,85,86,87,88,89,90,91,92,93,94,95,96,97] for detailed examination within the scope of this analysis.

### 3.2. Basic Characteristics of Included Studies

The seventeen investigations [83,84,85,86,87,88,89,90,91,92,93,94,95,96,97] implemented a retrospective approach, encompassing a cumulative patient population of 7830 individuals. The sizes of the patient cohorts exhibited significant variability, ranging from a minimum of 29 [11] to a maximum of 4100 [95] patients. A fundamental aspect of these investigations was the implementation of manual annotation, underscoring the indispensable role of human expertise within the research framework. The methodologies for validation adopted across these studies were bifurcated into either a train/test split [83,84,85,86,87,88,89,95,96] or cross-validation [11,12,90,91,92,93,94,97], with the criteria for annotation differing and encompassing evaluations by professionals such as experienced clinicians, radiologists, radiation oncologists, and oncologists (Table 1).

### 3.3. Characteristics of MRI

Magnetic field strengths span from 1.5 Tesla (T) [12,94] to 3T [11,84,91,92,95,97], with some studies encompassing both ranges [86,90]. This diversity underscores the extensive array of MRI technologies utilized in both the clinical and research domains. The MRI sequences investigated across these studies encompass T1 weighted (T1w), contrast-enhanced T1 weighted (T1c), T2 weighted (T2w), dynamic contrast enhanced (DCE), and a variety of specialized sequences, including Ktrans, T1 water, T2 water, fat-saturated T2 weighted (fs-T2W), and contrast-enhanced T1 weighted with fat saturation (fs-ce-T1W). With respect to hardware, the research references the utilization of apparatuses from foremost manufacturers, such as GE, Siemens, and Philips, among others, highlighting models like the GE Discovery MR 750w, Siemens Magnetom Skyra, Philips Achieva TX, and Siemens Aera (Table 2).

### 3.4. Characteristics and Performance of Preprocessing Techniques and DL Algorithms

Intensity normalization is prominently applied in a significant majority of investigations [11,12,83,84,85,86,87,89,90,91,92,93,96,97], underscoring its critical role in harmonizing the intensity scale across images to enhance algorithmic precision. Resolution adjustment, implemented selectively across studies [83,85,86,97], reflects a customized strategy to refine image resolution to meet specific analytical requisites. Image augmentation, a strategy designed to augment the diversity of training datasets, finds application in nearly all examined studies [94,95], evidencing its widespread adoption to bolster model resilience. Conversely, image cropping is utilized in various research endeavors [11,83,84,85,86,87,88,89,91,93], focusing model analysis on pertinent image regions (Table 3).

Research incorporating 3D input dimensions, such as those employing attention-guided Vnet [85], nnUNet [86], MMFNet [93], SC-DenseNet [95], and VoxResNet [96], illustrates a growing dependency on volumetric data to enhance accuracy in segmentation tasks, with nnUNet [86] achieving a notable Dice score of 0.88 across a dataset encompassing 600 samples. Conversely, 2D analyses persist in their prevalence, with CDDSA [87] attaining a Dice score of 0.92 on 114 samples, demonstrating the effectiveness of specialized 2D convolutional frameworks in distilling relevant features from intricate image datasets. The variation in training dataset sizes, ranging from a minimal 28 in studies utilizing CNNs [11] to a substantial 3285 in the context of SC-DenseNet [95], highlights the adaptability of deep learning frameworks to assimilate and learn from datasets of divergent scopes. Moreover, the application of distinct algorithms such as SICNet [83], ResU-Net [84], and T-U-Net [94] across various studies, with Dice scores fluctuating between 0.66 and 0.92, accentuates the diverse methodological tactics engaged within the domain (Table 3).

### 3.5. Quality Assessment

Figure S1 illustrates the quality assessments of the included studies conducted with the QUADAS-2 tool. Supplementary Table S6 details an analysis focusing on bias-related risks and applicability concerns, identifying ambiguous risks due to the exclusion of interval derivation in datasets in 10 (58.8%) of the studies [12,83,84,85,86,87,88,89,93,97], which may impact data interpretation. This criterion could influence the applicability and generalizability of the results from these studies.

Supplementary Table S7 reports a detailed evaluation of 17 studies using the CLAIM criteria, revealing an average CLAIM score of 27.35, equating to approximately 65.13% with a standard deviation of 3.86, and scores ranging from 23.00 to 33.00 out of a maximum of 42. The breakdown of average scores for CLAIM subsections indicates the quality of these studies as follows: title/abstract, 1.64/2 (82%); introduction, 2.00/2 (100%); methods, 18.18/28 (64.9%); results, 2.7/5 (54%); discussion, 1.94/2 (97%); and other information, 0.88/3 (29.4%). These results highlight the strengths and potential improvement areas in lung cancer research utilizing DL methodologies.

### 3.6. Efficacy of DL Model Segmentation of NPC on MRI 

**Figure 2:**
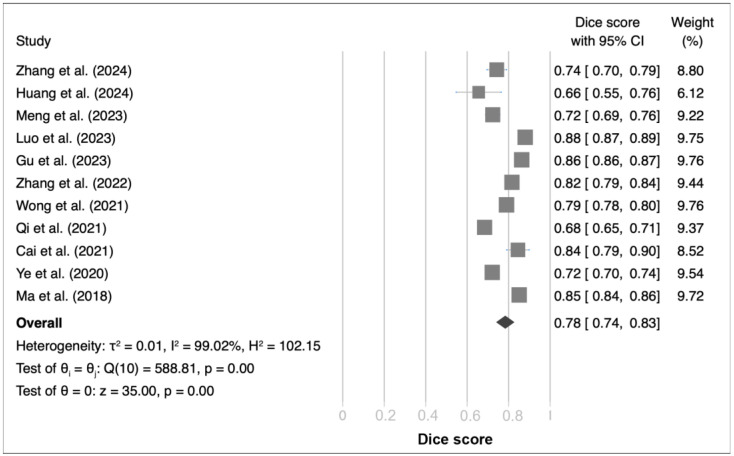
Forest plot of Dice scores for deep learning algorithms in independent datasets [12,83,84,85,86,87,88,91,92,93,94,97].

The investigation synthesized findings from 11 studies, each utilizing distinct datasets and DL models for segmentation tasks, and uncovered notable variations in Dice scores, which spanned from 66% to 84%. The consolidated outcomes produced a pooled Dice score of 78%, with a 95% confidence interval (CI) ranging from 74% to 83% (Figure 2). The Q test indicated substantial heterogeneity across the studies, as evidenced by a Q value of 588.81 with a significance level below 0.01. Further affirmation of this heterogeneity was provided by the Higgins I^2^ statistic, which reported a remarkably high degree of variability (I^2^ = 99.02%). Sensitivity analysis reinforced the reliability of these findings, affirming the statistical significance of the summary effect sizes even upon the sequential exclusion of individual studies from the analytical framework (Figure S2). Additionally, the funnel plot assessment of the 11 studies, coupled with Egger’s regression test, disclosed a *p*-value of 0.037, intimating the presence of publication bias within the examined corpus of studies (Figure S3). Nevertheless, subsequent analysis through subgrouping predicated on publication metrics failed to disclose any significant discrepancies (Figure S4).

Employing a sophisticated meta-analytic methodology, a three-level meta-analysis was undertaken to scrutinize potential moderating factors associated with DL models utilized in segmentation tasks. This meticulous examination included an extensive assessment of outcomes across numerous validation sets, augmented by clustering according to datasets to mitigate the impact of their repeated utilization. From an aggregation of 68 reported effects spanning 17 distinct studies, the mean Dice coefficient was calculated to be 76.4%, with a 95% CI ranging from 71.1% to 81.6%. The Q statistic analysis revealed an absence of significant heterogeneity, evidenced by a Q value of 55.4 (*p* = 0.821). Comparative evaluations employing Akaike and Bayesian information criteria highlighted a preference for the three-level model over conventional two-tiered approaches, highlighting its superior accuracy in representing the data structure. Further, variance analysis elucidated that 58.61% of the total variance was attributable to level 1 (sampling variance), with the residual variance delineated between within-dataset disparities (4.6e-8%) at level 2 and inter-dataset differences (41.39%) at level 3. This distribution of variability underscored significant inter-dataset variation, in contrast to negligible within-dataset discrepancies (Supplementary Table S5), reinforcing previously observed significant heterogeneity in meta-analyses of independent datasets. Meta-regression analysis probing factors such as dataset splitting, validation methodology, MRI sequence, algorithmic typology, training volume, and preprocessing approaches did not yield significant correlations with the segmentation efficacy of DL models. 

## 4. Discussion

The primary objective of this systematic review and meta-analysis was to assess the efficacy and accuracy of DL models, specifically in the segmentation of NPC in MRI. In the landscape of medical imaging, especially for conditions like NPC where precision in diagnosis and treatment planning is critical, the role of DL technologies marks a transformative potential. By focusing on MRI, this review targets an area where DL models can significantly leverage high-resolution images for better disease characterization.

### 4.1. Summary of Findings

Our comprehensive analysis revealed that DL models, particularly convolutional neural networks (CNNs), enhance the accuracy of NPC segmentation in MRI scans. The pooled analysis of Dice scores, a key metric for evaluating segmentation accuracy, included 11 studies with a total of 7830 patients or MRI scans. Using a random effects model, we calculated a pooled mean Dice score of 78% (95% confidence interval: 74% to 83%) across the included studies (Figure 2). The Dice score ranges from 0 to 1, with higher values indicating better segmentation accuracy. Heterogeneity among the studies was assessed using the Q test and the I^2^ statistic. The Q test indicated substantial heterogeneity across the studies (Q = 588.81, *p* < 0.01), and the I^2^ statistic revealed a high degree of variability (I^2^ = 99.02%). To explore the potential sources of heterogeneity, we conducted subgroup analyses and meta-regressions on variables such as publication status, MRI sequence, algorithm type, and preprocessing techniques (see Section 3.6 for details). The funnel plot assessment and Egger’s regression test (*p* = 0.037) suggested the presence of publication bias within the examined studies (Figure S3). However, further subgroup analysis based on publication status did not reveal any significant discrepancies (Figure S4). These findings underscore the effectiveness of DL models in improving NPC segmentation accuracy in MRI scans compared to traditional methods. The pooled mean Dice score of 78% indicates a moderate to high level of segmentation accuracy, highlighting the potential of DL models to enhance clinical decision making and treatment planning in NPC management. However, it is important to acknowledge the substantial heterogeneity observed among the included studies, which may stem from differences in patient populations, MRI acquisition protocols, and DL model architectures. 

### 4.2. Comparison with the Existing Literature

Previous reviews have extensively covered various applications of deep learning and machine learning for nasopharyngeal carcinoma (NPC) [98,99,100]. In the review by Li et al. [98], the authors briefly outline articles related to auto-segmentation using deep learning techniques. Ng et al. [99] presented a descriptive box plot in their study of auto-targeting, showing a median Dice score of 0.7530, which illustrates the current performance level in this field. Wang et al. [100] discussed the advantages and disadvantages of different imaging modalities. They noted that while CT images often lack sufficient soft tissue contrast, PET images provide excellent tumor visualization but fail to deliver accurate boundary information due to their low spatial resolution. Dual-modality PET-CT images, however, offer more valuable information for delineating tumor boundaries and assessing the extent of tumor invasion [101]. Despite its superior soft tissue contrast, MRI is considered the gold standard for staging and measuring target volume contours in NPC. However, identifying tumor margins on MRI can be challenging due to factors such as high variability, low contrast, and discontinuous soft tissue margins. While discussions on auto-segmentation using deep learning methods are present, there is a notable lack of comprehensive and quantitative analysis in the existing literature.

Compared to previous systematic reviews and meta-analyses on CT and MRI segmentation of nasopharyngeal cancer, our focused investigation into NPC segmentation exclusively using MRI technology represents a more specialized inquiry into this domain [14]. Our review not only corroborates the effectiveness of deep learning models in NPC segmentation, demonstrating a pooled Dice score of 78%, closely aligning with prior findings of 76% [14], but it also introduces several key differentiators that enhance the robustness and relevance of our conclusions. Notably, our review incorporates five additional studies from 2023 and 2024, broadening the evidence base. Our emphasis on MRI scans allowed for more nuanced data extraction and analysis, ensuring a deeper understanding of this specific imaging modality’s challenges and opportunities in NPC segmentation. Furthermore, we employed a two-pronged meta-analysis approach: a traditional two-level random effects model that addressed independent datasets and a novel three-level random effects model that accounted for all reported results across validation sets, effectively clustering by dataset. This methodology revealed significant heterogeneity among independent datasets, indicating the necessity for further research to explore the sources of this variability. Future studies are encouraged to expand the dataset to illuminate these findings further and comprehensively address the identified heterogeneity.

### 4.3. Strengths of Deep Learning Models

DL models handle complex, high-dimensional data, and are ideally suited for medical imaging tasks. Their strengths lie in rapid processing, high accuracy, and reproducibility, as demonstrated by models like nnU-Net [86] and CDDSA [87], which exhibited exemplary performance in our review. The nnU-Net (no-new-Net) [102] represents a significant stride in the application of deep learning for medical image segmentation, specifically highlighted in our review by its exceptional performance in NPC segmentation within MRI scans. Achieving a Dice score of 0.88, the nnU-Net not only demonstrates its robustness in precisely delineating the tumor boundaries in NPC but also underscores the model’s capability in handling the inherent complexities of medical imaging data. This performance is particularly noteworthy given the challenging nature of NPC, a cancer type characterized by its intricate anatomical location and the potential for subtle imaging signatures.

nnU-Net’s architecture is designed to automatically adapt to the segmentation task’s specifics, including optimizing its configuration to match the input data dimensions, preprocessing routines, and network architecture parameters. This adaptability is key to its success, enabling the nnU-Net to efficiently process the high-dimensional data typical of MRI scans, thereby ensuring high accuracy and reproducibility across different datasets and segmentation tasks. The model’s proficiency in capturing the nuanced details of NPC tumors from MRI without the need for extensive manual tuning or intervention represents a paradigm shift from traditional segmentation approaches, which are often time consuming and prone to inter- and intra-observer variability. By automating the segmentation process while maintaining, if not exceeding, the accuracy of manual methods, the nnUNet not only enhances diagnostic workflows but also paves the way for more personalized and timely treatment planning, leveraging the full potential of deep learning to improve patient care outcomes in oncology.

Comparing the three models, the nn-U-Net [86], CDDSA [87], and CNN [11], the studies using CDDSA and CNN demonstrated higher performance than the one using the nn-U-Net. All three studies utilized extensive preprocessing techniques such as intensity normalization, image augmentation, and image cropping. The study using the nn-U-Net [86] additionally employed resolution adjustment. It is important to note that the CNN study [11] from 2018 had a limited sample size of only 29 patients, which may affect the robustness and generalizability of their model’s performance. In contrast, the study using the nn-U-Net [86] included 1057 patients and performed external validation, demonstrating the most robust validation among the three. The CDDSA study [87] used 189 patients with internal validation, which can be considered decent. Moreover, the disentangle-based style augmentation technique utilized in the CDDSA study may have contributed to its high performance.

### 4.4. Limitations and Challenges

Despite the promising outcomes, our review faced limitations, including evident publication bias and significant study heterogeneity, which could influence the interpretability of our results. Moreover, while being the standard for comparison, the manual segmentation process introduces subjectivity and variability in outcomes. DL models, though superior, are not without challenges, including the need for extensive training data and the complexity of model tuning to achieve optimal performance.

### 4.5. Implications for Clinical Practice

Integrating DL models into clinical settings for NPC segmentation from MRI scans could revolutionize treatment planning and prognosis evaluation. The precision of DL-enhanced segmentation can lead to more accurate staging, targeted therapy, and monitoring strategies. However, for such integration to be successful and globally applicable, there is a critical need for standardization in DL model development, validation, and implementation across different healthcare contexts.

### 4.6. Future Research Directions

Future research should aim at developing more advanced DL models capable of accommodating the variability inherent in MRI data, including differences in imaging parameters and tumor presentation. Moreover, exploring DL applications beyond segmentation, such as in treatment response assessment and recurrence detection in NPC, could provide comprehensive tools for holistic disease management. This direction promises improvements in clinical outcomes and paves the way for personalized treatment approaches based on predictive analytics.

## 5. Conclusions

Our systematic review and meta-analysis have highlighted the effectiveness of deep learning (DL) models in improving the accuracy of nasopharyngeal carcinoma (NPC) segmentation in MRI scans, with a pooled mean Dice score of 78% (95% confidence interval: 74% to 83%), indicating a moderate to high segmentation accuracy in DL models. DL’s role in medical imaging, particularly for NPC, marks a significant advancement that matches the growing need for precision in medical diagnostics. However, the substantial heterogeneity and the presence of publication bias observed necessitate a careful interpretation of these results. They emphasize the need for further validation and standardization of DL models across varied clinical environments to confirm their effectiveness and consistency. While current deep learning models achieve moderate to high segmentation accuracy, further optimization and improvement of deep learning architectures are warranted. As we look forward, integrating DL into clinical practice is set to transform NPC management by equipping clinicians with more accurate tools, potentially enhancing personalized treatment and patient outcomes. Future research should extend the use of DL to other areas, such as treatment response monitoring and intraoperative imaging, maximizing the benefits of this technology in cancer care.

## Figures and Tables

**Table 1:** Basic characteristics.

First Author	Study Design	Patients	Series (Train/Valid/Test)	Reference	Validation	Data Source	Indicator Standard
Zhang et al. (2024) [83]	Retrospective	130	130 (90/15/25)	Manual	Train/Test	Guangdong Provincial People’s Hospital	Experienced clinician
Huang et al. (2024) [84]	Retrospective	96	96 (76/10/10)	Manual	Train/Test	Cancer Hospital Chinese Academy of Medical Sciences, Shenzhen Hospital.	Two radiologists
Meng et al. (2023) [85]	Retrospective	161	161 (129/0/32)	Manual	Train/Test	Cancer Hospital	Radiation oncologists
Luo et al. (2023) [86]	Retrospective	1057	1057 (600/259/198)	Manual	Train/Test	Southern Medical University, West China Hospital, Sichuan Provincial People’s Hospital, Anhui Provincial Hospital, Sichuan Cancer Hospital	Two oncologists
Gu et al. (2023) [87]	Retrospective	189	189 (114/0/75)	Manual	Train/Test	Sichuan Provincial People’s Hospital, West China Hospital	Radiation oncologists
Zhang et al. (2022) [88]	Retrospective	93	93 (75/9/9)	Manual	Train/Test	Sun Yat-sen University	NR
Liu et al. (2022) [89]	Retrospective	92	92 (74/9/9)	Manual	Train/Test	Sun Yat-sen University	NR
Li et al. (2022) [90]	Retrospective	754	754 (604/150/0)	Manual	Cross-validation	Sun Yat-sen University	Three radiologists
Wong et al. (2021) I [91]	Retrospective	404	404 (303/101/0)	Manual	Cross-validation	Joint Chinese University of Hong Kong	Expert
Wong et al. (2021) II [92]	Retrospective	201	201 (130/6/65)	Manual	Cross-validation	Joint Chinese University of Hong Kong	Expert
Qi et al. (2021) [93]	Retrospective	149	149 (119/30/0)	Manual	Cross-validation	Shandong Cancer Hospital Affiliated to Shandong University	Experienced radiologists
Cai et al. (2021) [94]	Retrospective	251	251 (226/25/0)	Manual	Cross-validation	Fudan University Shanghai Cancer Center	Radiation oncologist
Ye et al. (2020) [12]	Retrospective	44	44 (40/4/0)	Manual	Cross-validation	Panyu Central Hospital	Radiologist
Ke et al. (2020) [95]	Retrospective	4100	4100 (3285/411/404)	Manual	Train/Test	Sun Yat-sen University	Radiation oncologist
Lin et al. (2019) [96]	Retrospective	1021	1021 (715/103/203)	Manual	Train/Test	Sun Yat-sen University	Radiation oncologist
Ma et al. (2018) [97]	Retrospective	30	30 (29/1/0)	Manual	Cross-validation	West China Hospital	Radiation oncologist
Li et al. (2018) [11]	Retrospective	29	29 (28/1/0)	Manual	Cross-validation	Sun Yat-sen University	Radiologists

Abbreviations: NR, not recorded.

**Table 2:** Characteristics of MRI.

First Author	Tesla	Sequence	Hardware
Zhang et al. (2024) [83]	NR	T1c	NR
Huang et al. (2024) [84]	3T	DCE, Ktrans	GE Discovery MR 750w
Meng et al. (2023) [85]	NR	T2w	Siemens Magnetom Skyra
Luo et al. (2023) [86]	1.5T/3T	T1c	GE, Siemens, Philips
Gu et al. (2023) [87]	NR	T1w, T1c, T1 water, T2 water	NR
Zhang et al. (2022) [88]	NR	T1c	Siemens Aera
Liu et al. (2022) [89]	NR	T1c	Siemens Aera
Li et al. (2022) [90]	1.5T/3T	T1, T2, T1c	NR
Wong et al. (2021) I [91]	3T	T2w	Philips Achieva TX
Wong et al. (2021) II [92]	3T	T1W, fs-T2W, T1c and fs-ce-T1W	Philips Achieva TX
Qi et al. (2021) [93]	NR	T1, T2, T1c	NR
Cai et al. (2021) [94]	1.5T	T1, T2, T1c	GE, Milwaukee
Ye et al. (2020) [12]	1.5T	T1w, T2w	Siemens Avanto
Ke et al. (2020) [95]	3T	T1c	Trio Tim; SIEMENS, Achieva, PHILIPS; Discovery MR750; GE; Discovery MR750w; GE, USA
Lin et al. (2019) [96]	NR	T1, T2, T1c, T1w-fs	NR
Ma et al. (2018) [97]	3T	T1w	Philips Achieva
Li et al. (2018) [11]	3T	DCE	Magnetom Trio, Siemens

Abbreviations: NR, not recorded. DCE: dynamic contrast enhanced; fs: fat suppress; GE: General Electric.

**Table 3:** Characteristics and performance of preprocessing techniques and deep learning algorithms.

First Author	Intensity Normalization	Resolution Adjustment	Image Augmentation	Image Cropping	Training Size	Input Dimension	Algorithms	Dice Score
Zhang et al. (2024) [83]	Yes	Yes	Yes	Yes	90	2D/3D	SICNet	0.74
Huang et al. (2024) [84]	Yes	No	Yes	Yes	76	2D	ResU-Net	0.66
Meng et al. (2023) [85]	Yes	Yes	Yes	Yes	129	3D	Attention-guided Vnet	0.72
Luo et al. (2023) [86]	Yes	Yes	Yes	Yes	600	3D	nnUNet	0.88
Gu et al. (2023) [87]	Yes	No	Yes	Yes	114	2D	CDDSA	0.92
Zhang et al. (2022) [88]	No	No	Yes	Yes	75	2D	AttR2U-Net	0.82
Liu et al. (2022) [89]	Yes	No	Yes	Yes	74	2D	LW-UNet-3	0.81
Li et al. (2022) [90]	Yes	No	Yes	No	604	2D	NPCNet	0.73
Wong et al. (2021) I [91]	Yes	No	Yes	Yes	303	2D	CNN	0.79
Wong et al. (2021) II [92]	No	No	Yes	No	130	2D	U-net	0.73
Qi et al. (2021) [93]	Yes	No	Yes	Yes	149	3D	MMFNet	0.68
Cai et al. (2021) [94]	No	No	No	No	226	2D	T-U-Net	0.85
Ye et al. (2020) [12]	Yes	No	Yes	Yes	40	2D	DEU	0.72
Ke et al. (2020) [95]	No	No	No	No	3285	3D	SC-DenseNet	0.77
Lin et al. (2019) [96]	Yes	No	Yes	No	715	3D	VoxResNet	0.79
Ma et al. (2018) [97]	Yes	Yes	No	No	29	2D/3D	CNN	0.85
Li et al. (2018) [11]	Yes	No	Yes	Yes	28	2D	CNN	0.89

## Data Availability

Data are contained within the article and Supplementary Files.

## Supplementary Materials

The following supporting information can be downloaded at https://www.mdpi.com/article/10.3390/bioengineering11050504/s1, Figure S1: The results of QUADAS-2 quality assessment for the included studies. Figure S2: The results of a sensitivity analysis of deep learning algorithms in independent datasets using the one-study removal method. Figure S3: Funnel plot of Dice scores for deep learning algorithms in independent datasets. Figure S4: Forest plot of subgroup analysis deep learning algorithms in an independent dataset using publication status as moderator. Table S1: PRISMA-DTA Abstract Checklist. Table S2: PRISMA-DTA Checklist. Table S3: Keywords and search results in different databases. Table S4: Excluded articles and reasons. Table S5: Comparison of multilevel meta-analysis model clusters with datasets of segmentation Dice scores across all validation sets. Table S6: Quality assessment according to the Quality Assessment of Diagnostic Accuracy Studies 2 (QUADAS-2) criteria. Table S7: The Checklist for Artificial Intelligence in Medical Imaging scores.
