# Supplementary material for: Deep Learning for Nasopharyngeal Carcinoma Segmentation in Magnetic Resonance Imaging: A Systematic Review and Meta-Analysis
Source: Bioengineering (Basel). 2024 May 17;11(5):504. doi: 10.3390/bioengineering11050504 (PMC11118180; doi:10.3390/bioengineering11050504)
Supplement: Supplementary file 1 [file bioengineering-11-00504-s001.zip › bioengineering-2971327-supplementary.pdf]

Deep Learning for Nasopharyngeal Carcinoma Segmentation in MRI Imaging: A Systematic Review and Meta-Analysis

Chih-Keng Wang, Ting-Wei Wang, Ya-Xuan Yang, Yu-Te Wu

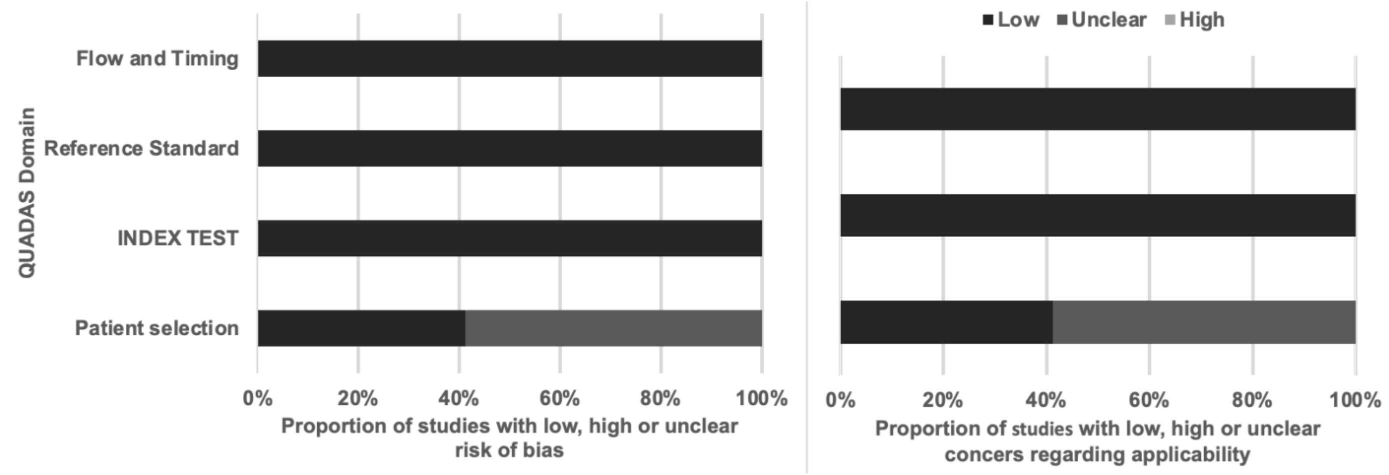

Figure S1. The results of QUADAS-2 quality assessment for included studies

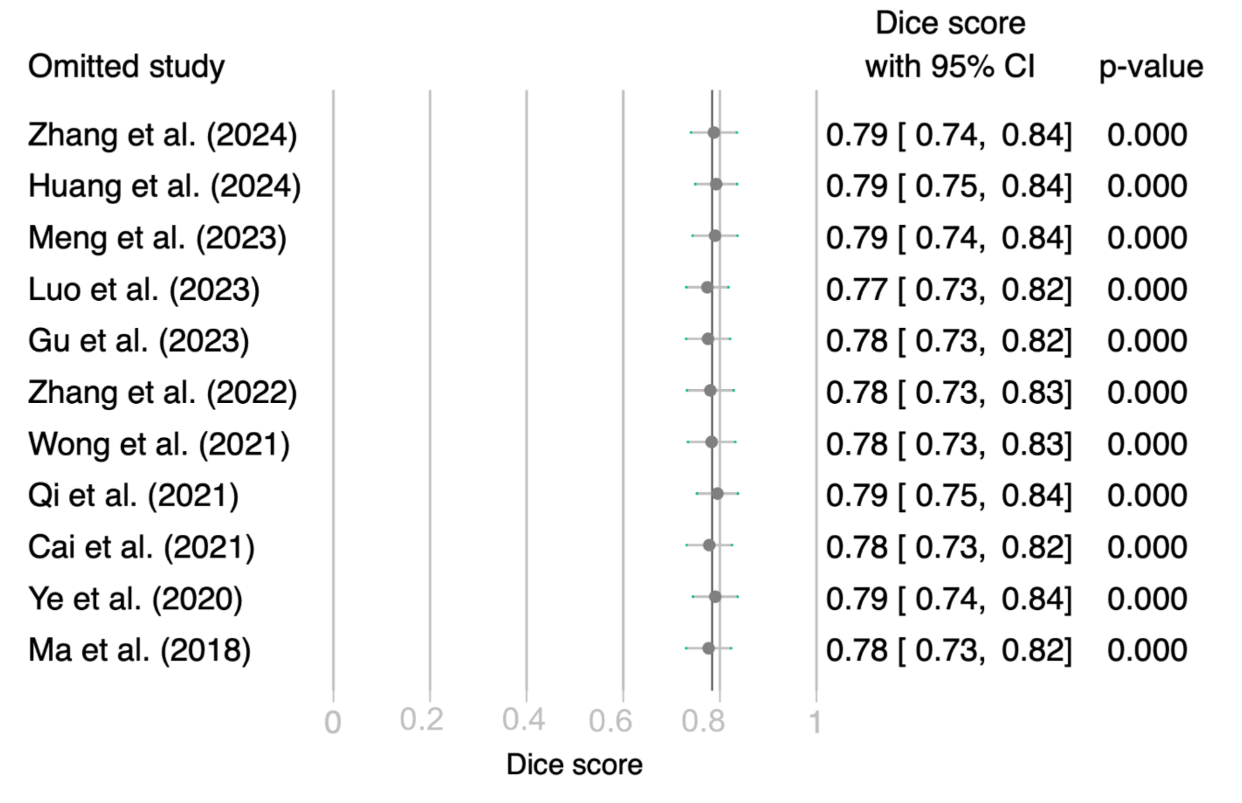

Figure S2. The results of a sensitivity analysis of deep learning algorithms in independent datasets using the one-study removal method.

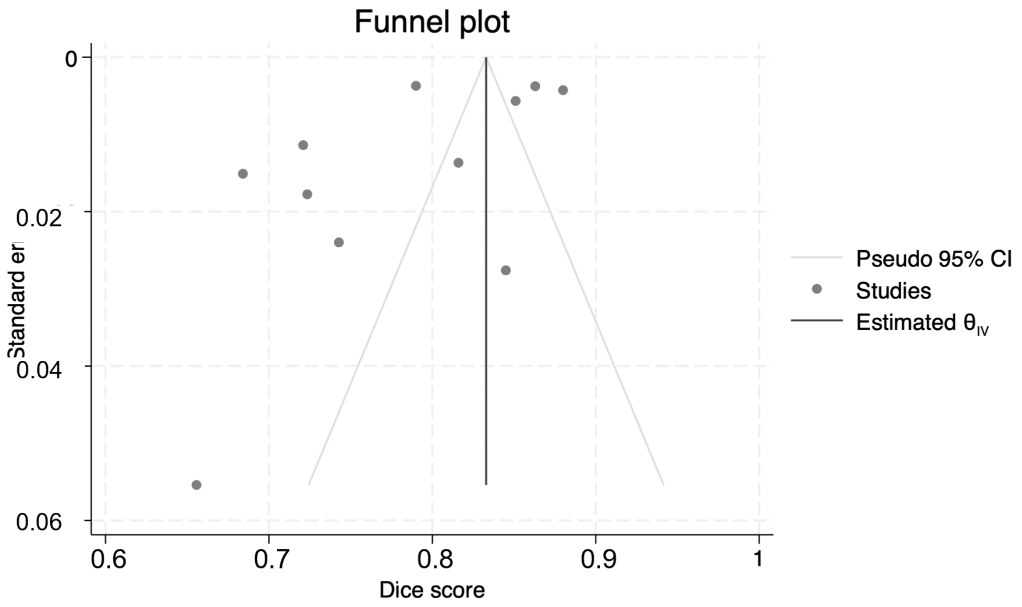

Figure S3. The funnel plot of Dice scores for deep learning algorithms in independent datasets. The *p* value of the Egger's test was 0.037 indicating present of publication bias.

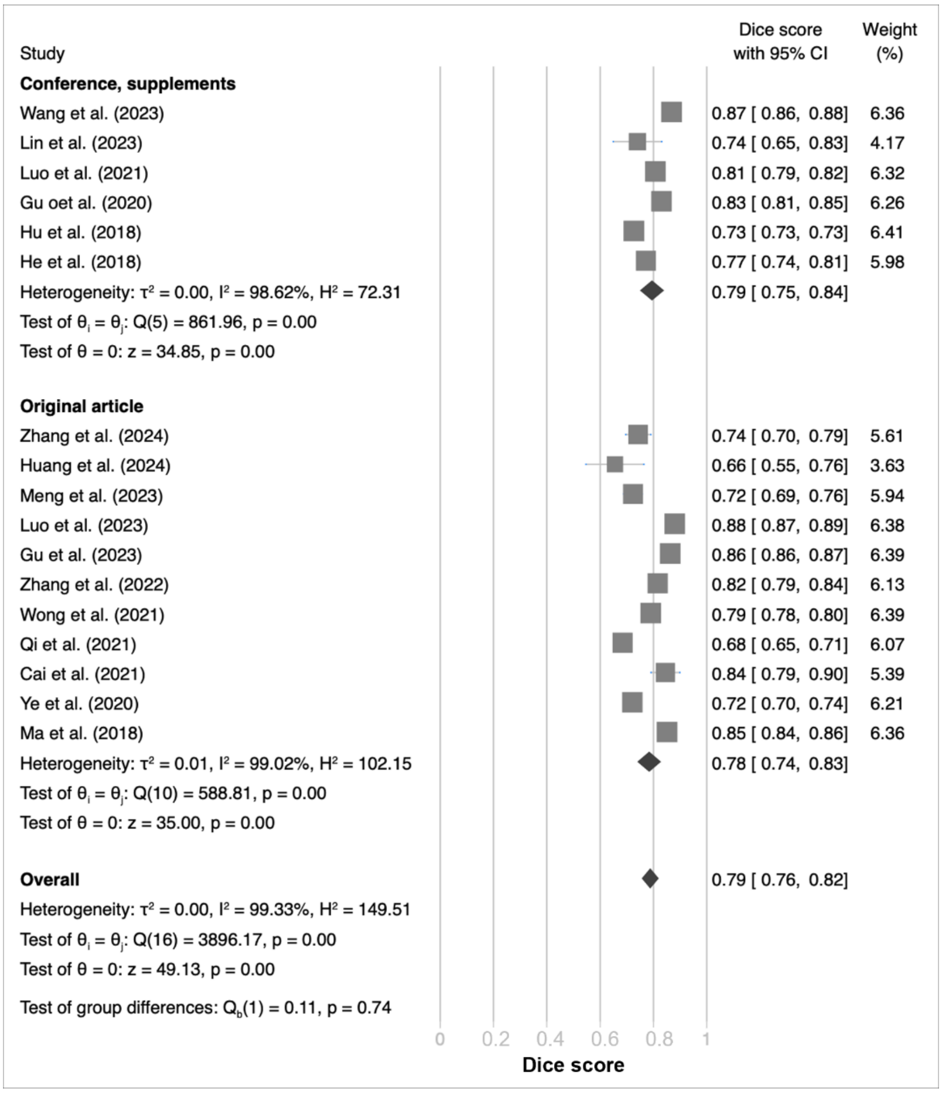

Figure S4. Forest plot of subgroup analysis deep learning algorithms in independent dataset using publication status as moderator

Table S1. PRISMA-DTA Abstract Checklist.

Section/Topic	Number	PRISMA-DTA for Abstracts Checklist Item	Reported on Page #
		TITLE and PURPOSE	
Title	1	Identify the report as a systematic review (+/- meta-analysis) of diagnostic test accuracy (DTA) studies.	1
Objectives	2	Indicate the research question, including components such as participants, index test, and target conditions.	1
		METHODS	
Eligibility criteria	3	Include study characteristics used as criteria for eligibility.	1
Information sources	4	List the key databases searched and the search dates.	1
Risk of bias & applicability	5	Indicate the methods of assessing risk of bias and applicability.	1
Synthesis of results	A1		1
		RESULTS	
Included studies	6	Indicate the number and type of included studies and the participants and relevant characteristics of the studies (including the reference standard).	1
Synthesis of results	7	Include the results for the analysis of diagnostic accuracy, preferably indicating the number of studies and participants. Describe test accuracy including variability; if meta-analysis was done, include summary results and confidence intervals.	1
		DISCUSSION	
Strengths and limitations	9	Provide a brief summary of the strengths and limitations of the evidence	1
Interpretation.	10	Provide a general interpretation of the results and the important implications.	1
		OTHER	
Funding	11	Indicate the primary source of funding for the review	NA
Registration	12	Provide the registration number and the registry name	NA

Adapted From: McInnes MDF, Moher D, et al. The PRISMA-DTA Group (2018). Preferred Reporting Items for a Systematic Review and Meta-analysis of Diagnostic Test Accuracy Studies: The PRISMA-DTA Statement. JAMA. 2018 Jan 23;319(4):388-396. doi: 10.1001/jama.2017.19163.

Table S2. PRISMA-DTA Checklist.

Section/Topic	Number	PRISMA-DTA for Abstracts Checklist Item	Reported on Page #
		TITLE and PURPOSE	
Title	1	Identify the report as a systematic review (+/- meta-analysis) of diagnostic test accuracy (DTA) studies.	1
Abstract	2	Abstract: See PRISMA-DTA for abstracts.	1
		INTRODUCTION	
Rationale	3	Describe the rationale for the review in the context of what is already known.	1-2
Clinical role of index test	D1	State the scientific and clinical background, including the intended use and clinical role of the index test, and if applicable, the rationale for minimally acceptable test accuracy (or minimum difference in accuracy for comparative design).	1-2
Objectives	4	Provide an explicit statement of question(s) being addressed in terms of participants, index test(s), and target condition(s).	1-2
		METHODS	
Protocol and registration	5	Indicate if a review protocol exists, if and where it can be accessed (e.g., Web address), and, if available, provide registration information including registration number.	2
Eligibility criteria	6	Specify study characteristics (participants, setting, index test(s), reference standard(s), target condition(s), and study design) and report characteristics (e.g., years considered, language, publication status) used as criteria for eligibility, giving rationale.	2

Information sources	7	Describe all information sources (e.g., databases with dates of coverage, contact with study authors to identify additional studies) in the search and date last searched.	2
Search	8	Present full search strategies for all electronic databases and other sources searched, including any limits used, such that they could be repeated.	2
Study selection	9	State the process for selecting studies (i.e., screening, eligibility, included in systematic review, and, if applicable, included in the meta-analysis).	2
Data collection process	10	Describe method of data extraction from reports (e.g., piloted forms, independently, in duplicate) and any processes for obtaining and confirming data from investigators.	2
Definitions for data extraction	11	Provide definitions used in data extraction and classifications of target condition(s), index test(s), reference standard(s) and other characteristics (e.g. study design, clinical setting).	2
Risk of bias and applicability	12	Describe methods used for assessing risk of bias in individual studies and concerns regarding the applicability to the review question.	3
Diagnostic accuracy measures	13	State the principal diagnostic accuracy measure(s) reported (e.g. sensitivity, specificity) and state the unit of assessment (e.g. per-patient, per-lesion).	3
Synthesis of results	14	Describe methods of handling data, combining results of studies and describing variability between studies. This could include, but is not limited to: a) handling of multiple definitions of target condition. b) handling of multiple thresholds of test positivity, c) handling multiple index test readers, d) handling of indeterminate test results, e) grouping and comparing tests, f) handling of different reference standards	3
Meta-analysis	D2	Report the statistical methods used for meta-analyses, if performed.	3
Additional analyses	16	Describe methods of additional analyses (e.g., sensitivity or subgroup analyses, meta-regression), if done, indicating which were pre-specified.	3
		RESULTS	
Study selection	17	Provide numbers of studies screened, assessed for eligibility, included in the review (and included in meta-analysis, if applicable) with reasons for exclusions at each stage, ideally with a flow diagram	4
Study characteristics	18	For each included study provide citations and present key characteristics including: a) participant characteristics (presentation, prior testing), b) clinical setting, c) study design, d) target condition definition, e) index test, f) reference standard, g) sample size, h) funding sources	4-7
Risk of bias and applicability	19	Present evaluation of risk of bias and concerns regarding applicability for each study.	7
Results of individual studies	20	For each analysis in each study (e.g. unique combination of index test, reference standard, and positivity threshold) report 2x2 data (TP, FP, FN, TN) with estimates of diagnostic accuracy and confidence intervals, ideally with a forest or receiver operator characteristic (ROC) plot.	NA
Synthesis of results	21	Describe test accuracy, including variability; if meta-analysis was done, include results and confidence intervals	7-8
Additional analysis	23	Give results of additional analyses, if done (e.g., sensitivity or subgroup analyses, meta-regression; analysis of index test: failure rates, proportion of inconclusive results, adverse events).	7-8
		DISCUSSION	
Summary of evidence	24	Summarize the main findings including the strength of evidence	9-10
Limitations	25	Discuss limitations from included studies (e.g. risk of bias and concerns regarding applicability) and from the review process (e.g. incomplete retrieval of identified research).	9-10
Conclusions	26	Provide a general interpretation of the results in the context of other evidence. Discuss implications for future research and clinical practice (e.g. the intended use and clinical role of the index test)	10
		OTHER	
Funding	27	For the systematic review, describe the sources of funding and other support and the role of the funders	11

Adapted From: McInnes MDF, Moher D, et al. The PRISMA-DTA Group (2018). Preferred Reporting Items for a Systematic Review and Meta-analysis of Diagnostic Test Accuracy Studies: The PRISMA-DTA Statement. JAMA. 2018 Jan 23;319(4):388-396. doi: 10.1001/jama.2017.19163.

Table S3. Keywords and search results in different database

Database	Keyword	Date	Results
PubMed	((Nasopharyngeal Neoplasms OR Nasopharyngeal Cancer OR Nasopharyngeal Carcinoma OR Nasopharyngeal Tumors) AND (MRI OR magnetic resonance imaging OR MR) AND (segmentation OR contouring OR delineation) AND (deep learning OR convolutional neural networks OR CNN))	2024/03/20	36
Embase	((Nasopharyngeal Neoplasms OR Nasopharyngeal Cancer OR Nasopharyngeal Carcinoma OR Nasopharyngeal Tumors) AND (MRI OR magnetic resonance imaging OR MR) AND (segmentation OR contouring OR delineation) AND (deep learning OR convolutional neural networks OR CNN))	2024/03/20	72
Web of Science	((Nasopharyngeal Neoplasms OR Nasopharyngeal Cancer OR Nasopharyngeal Carcinoma OR Nasopharyngeal Tumors) AND (MRI OR magnetic resonance imaging OR MR) AND (segmentation OR contouring OR delineation) AND (deep learning OR convolutional neural networks OR CNN))	2024/03/20	68

Table S4. Excluded article and reason.

Title	Exclusion reason	
Investigation of autosegmentation techniques on T2-weighted MRI for off-line dose reconstruction in MR-linac workflow for head and neck cancers [27]	Outcome insufficient for quantitative analysis	No confidence interval or interquartile rage reported
Full-scale attention network for automated organ segmentation on head and neck CT and MR images [28]	Outcome not related to interest	No nasopharyngeal cancer segmentation reported
Deep Learning-Based Multi-Modality Segmentation of Primary Gross Tumor Volume in CT and MRI for Nasopharyngeal Carcinoma [29]	Outcome insufficient for quantitative analysis	No confidence interval or interquartile rage reported
DCTR U-Net: automatic segmentation algorithm for medical images of nasopharyngeal cancer in the context of deep learning [30]	Outcome insufficient for quantitative analysis	No confidence interval or interquartile rage reported
Automatic Head-and-Neck Tumor Segmentation in MRI via an End-to-End Adversarial Network [31]	Outcome insufficient for quantitative analysis	No confidence interval or interquartile rage reported
Automatic detection and recognition of nasopharynx gross tumour volume (GTVnx) by deep learning for nasopharyngeal cancer radiotherapy through magnetic resonance imaging [32]	Outcome not related to interest	Dice score not reported
Advancing Delineation of Gross Tumor Volume Based on Magnetic Resonance Imaging by Performing Source-Free Domain Adaptation in Nasopharyngeal Carcinoma [33]	Conference	
Young oncologists benefit more than experts from deep learning-based organs-at-risk contouring modeling in nasopharyngeal carcinoma radiotherapy: A multi-institution clinical study exploring working experience and institute group style factor [34]	Outcome not related to interest	No nasopharyngeal cancer segmentation reported

Objective Boundary Generation for Gross Target Volume and Organs at Risk Using 3D Multi-Modal Medical Images [35]	Outcome insufficient for quantitative analysis	No confidence interval or interquartile rage reported
Deep Learning-Based Synthesis of Contrast-Enhanced MRI for Automated Delineation of Primary Gross Tumor Volume in Radiotherapy of Nasopharyngeal Carcinoma [36]	Supplement	
Automatic tumor segmentation and metachronous single-organ metastasis prediction of nasopharyngeal carcinoma patients based on multi-sequence magnetic resonance imaging [37]	Outcome not related to interest	Dice score not reported
MSU-Net: Multi-scale Sensitive U-Net based on pixel-edge-region level collaborative loss for nasopharyngeal MRI segmentation [38]	Outcome insufficient for quantitative analysis	No confidence interval or interquartile rage reported
Dual-feature Fusion Attention Network for Small Object Segmentation [39]	Outcome insufficient for quantitative analysis	No confidence interval or interquartile rage reported
Automated Segmentation of Nasopharyngeal Carcinoma Based on Dual-Sequence Magnetic Resonance Imaging Using Self-supervised Learning [40]	Outcome insufficient for quantitative analysis	No confidence interval or interquartile rage reported
Application of Artificial Intelligence in Radiotherapy of Nasopharyngeal Carcinoma with Magnetic Resonance Imaging [41]	Retracted	
Patient-Specific Daily Updated Deep Learning Auto-Segmentation for MRI-Guided Adaptive Radiotherapy [42]	Outcome not related to interest	No nasopharyngeal cancer segmentation reported
MRI-guided Automated Delineation of Gross Tumor Volume for Nasopharyngeal Carcinoma using Deep Learning [43]	Outcome insufficient for quantitative analysis	No confidence interval or interquartile rage reported
Multiscale Local Enhancement Deep Convolutional Networks for the Automated 3D Segmentation of Gross Tumor Volumes in Nasopharyngeal Carcinoma: A Multi-Institutional Dataset Study [44]	Not MRI	
SeqSeg: A sequential method to achieve nasopharyngeal carcinoma segmentation free from background dominance [45]	Outcome insufficient for quantitative analysis	No confidence interval or interquartile rage reported
Convolutional Neural Network in Evaluation of Radiotherapy Effect for Nasopharyngeal Carcinoma [46]	Outcome insufficient for quantitative analysis	No confidence interval or interquartile rage reported
Multidimensional CNN-Based Deep Segmentation Method for Tumor Identification [47]	Retracted	
NPCFORMER: AUTOMATIC NASOPHARYNGEAL CARCINOMA SEGMENTATION BASED ON BOUNDARY ATTENTION AND GLOBAL POSITION CONTEXT ATTENTION [48]	Outcome insufficient for quantitative analysis	No confidence interval or interquartile rage reported
A multi-perspective information aggregation network for automatedT-staging detection of nasopharyngeal carcinoma [49]	Outcome insufficient for quantitative analysis	No confidence interval or interquartile rage reported

Patient-specific daily updated deep learning auto-segmentation for MRI-guided adaptive radiotherapy [50]	Outcome not related to interest	No nasopharyngeal cancer segmentation reported
Virtual Contrast-Enhanced Magnetic Resonance Images Synthesis for Patients With Nasopharyngeal Carcinoma Using Multimodality-Guided Synergistic Neural Network [51]	Outcome insufficient for quantitative analysis	No confidence interval or interquartile rage reported
Anatomical Partition-Based Deep Learning: An Automatic Nasopharyngeal MRI Recognition Scheme [52]	Outcome not related to interest	No nasopharyngeal cancer segmentation reported
Quantitative Comparisons of Deep-learning-based and Atlas-based Au-to-segmentation of the Intermediate Risk Clinical Target Volume for Nasopharyngeal Carcinoma [53]	Not MRI	
The contrast-enhanced MRI can be substituted by unenhanced MRI in identifying and automatically segmenting primary nasopharyngeal carcinoma with the aid of deep learning models: An exploratory study in large-scale population of endemic area [54]	Outcome not related to interest	Dice score not reported
A Novel Fully Automated MRI-Based Deep-Learning Method for Segmentation of Nasopharyngeal Carcinoma Lymph Nodes [55]	Outcome not related to interest	lymph node segmentation
A Preliminary Experience of Implementing Deep-Learning Based Auto-Segmentation in Head and Neck Cancer: A Study on Real-World Clinical Cases [56]	Outcome not related to interest	No nasopharyngeal cancer segmentation reported
AccuLearning: A User-Friendly Deep Learning Auto-Segmentation Platform for Radiotherap [57]	Outcome not related to interest	No nasopharyngeal cancer segmentation reported
Convolutional Neural Network Intelligent Segmentation Algorithm-Based Magnetic Resonance Imaging in Diagnosis of Nasopharyngeal Carcinoma Foci [58]	Outcome insufficient for quantitative analysis	No clear data splitting sample reported
DA-DSUnet: Dual Attention-based Dense SU-net for automatic head-and-neck tumor segmentation in MRI images [8]	Outcome insufficient for quantitative analysis	No confidence interval or interquartile rage reported
Artificial intelligence-based bone-enhanced magnetic resonance image-a computed tomography/magnetic resonance image composite image modality in nasopharyngeal carcinoma radiotherapy [59]	Outcome insufficient for quantitative analysis	No confidence interval or interquartile rage reported
MRI-Only Radiotherapy Planning for Nasopharyngeal Carcinoma Using Deep Learning [60]	Outcome not related to interest	Dice score not reported
Efficient Semi-supervised Gross Target Volume of Nasopharyngeal Carcinoma Segmentation via Uncertainty Rectified Pyramid Consistency [61]	Conference	
Head-Neck Cancer Delineation [62]	Review	

DCNet: Densely Connected Deep Convolutional Encoder-Decoder Network for Nasopharyngeal Carcinoma Segmentation [63]	Outcome insufficient for quantitative analysis	No confidence interval or interquartile rage reported
A deep learning approach to segmentation of nasopharyngeal carcinoma using computed tomography [64]	Not MRI	
Sequential and Iterative Auto-Segmentation of High-Risk Clinical Target Volume for Radiotherapy of Nasopharyngeal Carcinoma in Planning CT Images [65]	Not MRI	
Auto-segmentation of organs at risk for head and neck radiotherapy planning: From atlas-based to deep learning methods [66]	Review	
Coarse-to-fine Nasopharyngeal Carcinoma Segmentation in MRI via Multi-stage Rendering [67]	Outcome insufficient for quantitative analysis	No confidence interval or interquartile rage reported
Automatic Segmentation Of Nasopharyngeal Carcinoma On MR Images: A Single-Institution Experience [68]	Supplement	
automatic segmentation of nasopharyngeal carcinoma: a solution for single institution [69]	Supplement	
Image segmentation of nasopharyngeal carcinoma using 3D CNN with long-range skip connection and multi-scale feature pyramid [70]	Outcome insufficient for quantitative analysis	No confidence interval or interquartile rage reported
Combining Images and Clinical Diagnostic Information to Improve Automatic Segmentation of Nasopharyngeal Carcinoma Tumors on MR Images [71]	Supplement	
A deep learning based auto-segmentation for GTVs on NPC MR images [72]	Not MRI	
Deep-learning automatic delineation of primary tumour volume in nasopharyngeal carcinoma on T2W fat-suppressed MR images [73]	Outcome insufficient for quantitative analysis	No confidence interval or interquartile rage reported
Nasopharyngeal carcinoma segmentation based on enhanced convolutional neural networks using multi-modal metric learning [74]	Not MRI	
Achieving Accurate Segmentation of Nasopharyngeal Carcinoma in MR Images Through Recurrent Attention [75]	Outcome insufficient for quantitative analysis	No confidence interval or interquartile rage reported
Automatic Tumor Segmentation with Deep Convolutional Neural Networks for Radiotherapy Applications [76]	Outcome insufficient for quantitative analysis	No confidence interval or interquartile rage reported
Development and Validation of A Deep Learning Algorithm for Automated Delineation of Primary Tumor for Nasopharyngeal Carcinoma from Multimodal Magnetic Resonance Images [77]	Supplement	
A DISCRIMINATIVE LEARNING BASED APPROACH FOR AUTOMATED NASOPHARYNGEAL CARCINOMA	Not MRI	

SEGMENTATION LEVERAGING MULTI-MODALITY SIMILARITY METRIC LEARNING [78]		
A 2.5D Cancer Segmentation for MRI Images Based on U-Net[79]	Conference	
A 3D Dual Path U-Net of Cancer Segmentation Based on MRI [80]	Conference	
Deep Deconvolutional Neural Network for Target Segmentation of Nasopharyngeal Cancer in Planning Computed Tomography Images [81]	Outcome insufficient for quantitative analysis	No confidence interval or interquartile rage reported
Automatic Nasopharyngeal Carcinoma Segmentation in MR Images with Convolutional Neural Networks [82]	Conference	

Table S5. Comparison of multilevel meta-analysis model clusters with datasets of segmentation Dice scores across all validation sets.

	df	AIC	BIC	Log likelihood	LRT	p-value
Three-level model	3	-107.53	-100.96	56.76		
Within-studies variance constrained	2	-109.53	-105.15	56.76	0.00	1.00
Between-studies variance constrained	2	-97.15	-92.77	50.57	12.38	<0.01
Both variance components constrained	1	-93.35	-91.16	47.67	18.18	<0.01

Table S6. Quality assessment according to the Quality Assessment of Diagnostic Accuracy Studies 2 (QUADAS-2) criteria

Source	Risk of bias											Concern of applicability		
	**Patient selection:**			**INDEX TEST**		**Reference Standard**		**Flow and Timing**						
	**Consecutive**	**Case-control**	**Inappropriate exclusions**	**Blind to reference standard**	**Threshold prespecified**	**Correctly classify the target condition**	**Blind to index test**	**Appropriate interval**	**Receive a reference standard**	**Same reference standard**	**All patients analyzed**	**Patient selection**	**INDEX TEST**	**Reference Standard**
Zhang et al. (2024) [83]	Unclear	No	No	Yes	Yes	Yes	Yes	Yes	Yes	Yes	Yes	Unclear	Low	Low
Huang et al. (2024) [84]	Unclear	No	No	Yes	Yes	Yes	Yes	Yes	Yes	Yes	Yes	Unclear	Low	Low
Meng et al. (2023) [85]	Unclear	No	No	Yes	Yes	Yes	Yes	Yes	Yes	Yes	Yes	Unclear	Low	Low
Luo et al. (2023) [86]	Unclear	No	No	Yes	Yes	Yes	Yes	Yes	Yes	Yes	Yes	Unclear	Low	Low
Gu et al. (2023) [87]	Unclear	No	No	Yes	Yes	Yes	Yes	Yes	Yes	Yes	Yes	Unclear	Low	Low
Zhang et al. (2022) [88]	Unclear	No	No	Yes	Yes	Yes	Yes	Yes	Yes	Yes	Yes	Unclear	Low	Low
Liu et al. (2022) [89]	Unclear	No	No	Yes	Yes	Yes	Yes	Yes	Yes	Yes	Yes	Unclear	Low	Low
Li et al. (2022) [90]	Yes	No	No	Yes	Yes	Yes	Yes	Yes	Yes	Yes	Yes	Low	Low	Low
Wong et al. (2021) I [91]	Yes	No	No	Yes	Yes	Yes	Yes	Yes	Yes	Yes	Yes	Low	Low	Low
Wong et al. (2021) II [92]	Yes	No	No	Yes	Yes	Yes	Yes	Yes	Yes	Yes	Yes	Low	Low	Low
Qi et al. (2021) [93]	Unclear	No	No	Yes	Yes	Yes	Yes	Yes	Yes	Yes	Yes	Unclear	Low	Low
Cai et al. (2021) [94]	Yes	No	No	Yes	Yes	Yes	Yes	Yes	Yes	Yes	Yes	Low	Low	Low
Ye et al. (2020) [12]	Unclear	No	No	Yes	Yes	Yes	Yes	Yes	Yes	Yes	Yes	Unclear	Low	Low
Ke et al. (2020) [95]	Yes	No	No	Yes	Yes	Yes	Yes	Yes	Yes	Yes	Yes	Low	Low	Low
Lin et al. (2019) [96]	Yes	No	No	Yes	Yes	Yes	Yes	Yes	Yes	Yes	Yes	Low	Low	Low
Ma et al. (2018) [97]	Unclear	No	No	Yes	Yes	Yes	Yes	Yes	Yes	Yes	Yes	Unclear	Low	Low
Li et al. (2018) [11]	Yes	No	No	Yes	Yes	Yes	Yes	Yes	Yes	Yes	Yes	Low	Low	Low

Table S7. The Checklist for Artificial Intelligence in Medical Imaging scores.

Source	Title/Abstract	Introduction	Methods							Results		Discussion	Other Information	Total Score
			Study design	Data	Ground truth	Data preparation	Model	Training	Evaluation	Data	Model performance			
	(2)	(2)	(2)	(7)	(5)	(3)	(3)	(3)	(5)	(2)	(3)	(2)	(3)	(42)
Zhang et al. (2024) [83]	1	2	2	4	1	2	1	2	4	0	2	2	1	24
Huang et al. (2024) [84]	1	2	2	4	2	2	2	2	4	0	2	2	1	26
Meng et al. (2023) [85]	2	2	2	4	1	2	3	2	4	0	2	2	1	27
Luo et al. (2023) [86]	2	2	2	5	3	2	2	3	5	2	2	2	1	33
Gu et al. (2023) [87]	2	2	2	4	1	2	1	2	2	0	2	2	1	23
Zhang et al. (2022) [88]	2	2	2	3	1	2	1	1	4	0	3	2	1	24
Liu et al. (2022) [89]	2	2	2	4	0	2	1	1	3	0	2	2	1	22
Li et al. (2022) [90]	1	2	2	6	3	2	3	2	4	1	2	2	0	30
Wong et al. (2021) I [91]	2	2	2	6	4	2	2	2	4	1	2	2	1	32
Wong et al. (2021) II [92]	2	2	2	6	4	2	2	2	4	1	2	2	1	32
Qi et al. (2021) [93]	1	2	2	4	3	2	1	1	3	0	2	2	1	24
Cai et al. (2021) [94]	1	2	2	4	2	2	2	1	4	1	2	2	0	25
Ye et al. (2020) [12]	1	2	2	3	3	2	2	2	3	0	2	1	1	24
Ke et al. (2020) [95]	2	2	2	7	4	2	1	1	4	2	3	2	1	33
Lin et al. (2019) [96]	2	2	2	5	4	2	2	2	4	2	2	2	1	32
Ma et al. (2018) [97]	2	2	2	4	2	2	1	2	4	0	2	2	1	26
Li et al. (2018) [11]	2	2	2	4	4	2	2	1	4	0	2	2	1	28
